# Bioactive coating of zirconia toughened alumina ceramic implants improves cancellous osseointegration

**DOI:** 10.1038/s41598-019-53094-5

**Published:** 2019-11-13

**Authors:** Anne-Marie Pobloth, Max J. Mersiowsky, Luisa Kliemt, Hanna Schell, Anke Dienelt, Berit M. Pfitzner, Rainer Burgkart, Rainer Detsch, Dag Wulsten, Aldo R. Boccaccini, Georg N. Duda

**Affiliations:** 1 Julius Wolff Institut, Charité – Universitätsmedizin Berlin, Augustenburger Platz 1, 13353 Berlin, Germany; 2 Berlin-Brandenburg Center for Regenerative Therapies, Charité – Universitätsmedizin Berlin, Augustenburger Platz 1, 13353 Berlin, Germany; 3 Institut für Pathologie, Charité - Universitätsmedizin Berlin, Charitéplatz 1, 10117 Berlin, Germany; 4 Clinic of Orthopedics and Sports Orthopedics, Klinikum Rechts der Isar, Technische Universität München, Ismaninger Straße 22, D-81675 München, Germany; 5 Institute of Biomaterials, University of Erlangen-Nuremberg, Cauerstr. 6, 91058 Erlangen, Germany; 6 Center for Musculoskeletal Surgery, Charité - Universitätsmedizin Berlin, Augustenburger Platz 1, 13353 Berlin, Germany

**Keywords:** Translational research, Biomedical materials

## Abstract

Bioactive coatings have the potential to improve the bony integration of mechanically loaded orthopedic ceramic implants. Using the concept of mimicking the natural bone surface, four different coatings of varying thickness on a zirconia toughened alumina (ZTA) ceramic implant were investigated regarding their osseointegration in a drill-hole model in sheep. The hypothesis that a bioactive coating of ZTA ceramics would facilitate cancellous bone integration was investigated. The bioactive coatings consisted of either a layer of covalently bound multi phosphonate molecules (chemical modification = CM), a nano hydoxyapatite coating (HA), or two different bioactive glass (BG) coatings in micrometer thickness, forming a hydroxyl-carbonate apatite layer on the implant surface *in vivo* (dip-coated 45S5 = DipBG; sol-gel 70S30C = SGBG). Coated surfaces were characterized by scanning electron microscopy and X-ray photoelectron spectroscopy. After 12 weeks, osseointegration was evaluated via mechanical push-out testing and histology. HA enhanced the maximum push-out force (HA: mean 3573.85 ± 1119.91 N; SGBG: mean 1691.57 ± 986.76 N; p = 0.046), adhesive shear strength (HA: mean 9.82 ± 2.89 MPA; SGBG: mean 4.57 ± 2.65 MPA; p = 0.025), and energy release rate (HA: mean 3821.95 ± 1474.13 J/mm^2^; SGBG: mean 1558.47 ± 923.47 J/mm^2^; p = 0.032) compared to SGBG. The implant-bone interfacial stiffness increased by CM compared to SGBG coating (CM: mean 6258.06 ± 603.80 N/mm; SGBG: mean 3565.57 ± 1705.31 n/mm; p = 0.038). Reduced mechanical osseointegration of SGBG coated implants could be explained histologically by a foreign body reaction surrounding the implants.

## Introduction

Zirconia toughened alumina (ZTA) ceramic material are widely used in implants for joint replacement and joint reconstruction surgeries^1,2^. With the increasing number of young and active patients, expectations on the long-term function of primary joint replacement have increased substantially^3,4^. Further, active lifestyles coincide with increased life expectancy, clearly emphasizing the necessity of long-term implant stability and durability. In comparison to conventional implant materials such as polyethylene or metal, ZTA ceramics possess a high biocompatibility, wettability, and the lowest abrasion and wear debris production in comparison to other bearings, fulfilling the lifetime demands of highly active patients^5^. Recently, ZTA ceramics showed their potential to reduce the risk of revision surgery due to infection in comparison to metals and polymers^6^. ZTA ceramics are bioinert and enable only direct bone contact (contact osteogenesis), but no direct bonding to the surrounding bone. In contrast, bioactive materials such as bioactive glasses (BG) or hydroxyapatite (HA) enable direct bonding osteogenesis^7,8^. Thus, the strategy to combine the benefits of ZTA ceramic substrate with a bioactive coating could extend the use of ZTA ceramic to implant areas where direct bone bonding is necessary.

Therefore, four bioactive coatings, mimicking the natural bone surface, were investigated for the first time on a rough textured cylindrical ZTA ceramic implant for their ability to enhance the biological and mechanical integration into cancellous bone. The coating thickness ranged from nanometer to micrometer size. The textured surface structure decreases the implant’s stiffness at the interface and limits the bone shielding effect generally caused by orthopedic implants^9^. This results in a better stress distribution across the surrounding bone^10^ and an increased surface area for bone anchoring and ingrowth. The coatings under investigation were i) a chemical modification (CM) of the surface by covalent bound of multi phosphonate molecules (CM group), thus mimicking an HA surface, ii) a thin nano HA coating (HA group), and iii) two different BG coatings. For these two coatings a BG of 45S5 composition applied by a dipping technique (DipBG group) or a BG synthesized by sol-gel of composition 70S30C (SGBG group) was used. BG forms in contact with body fluids a hydroxy-carbonate apatite layer with a strong interfacial bonding with bone^11^. Non-coated (NC group) implants were used as a control. We hypothesized that the specific surface modification via bioactive coating in various thicknesses would improve the biological integration of textured ZTA ceramic implants into trabecular bone. To prove this hypothesis, a large animal model with drill hole defects in the epi-metaphyseal region of long bones was used. Implant integration was analyzed in a biomechanical push out test, histomorphometrical analyses, and descriptive histological evaluation of the bone implant interface 12 weeks after surgery.

## Materials and Methods

### Implants

**Figure 1:**
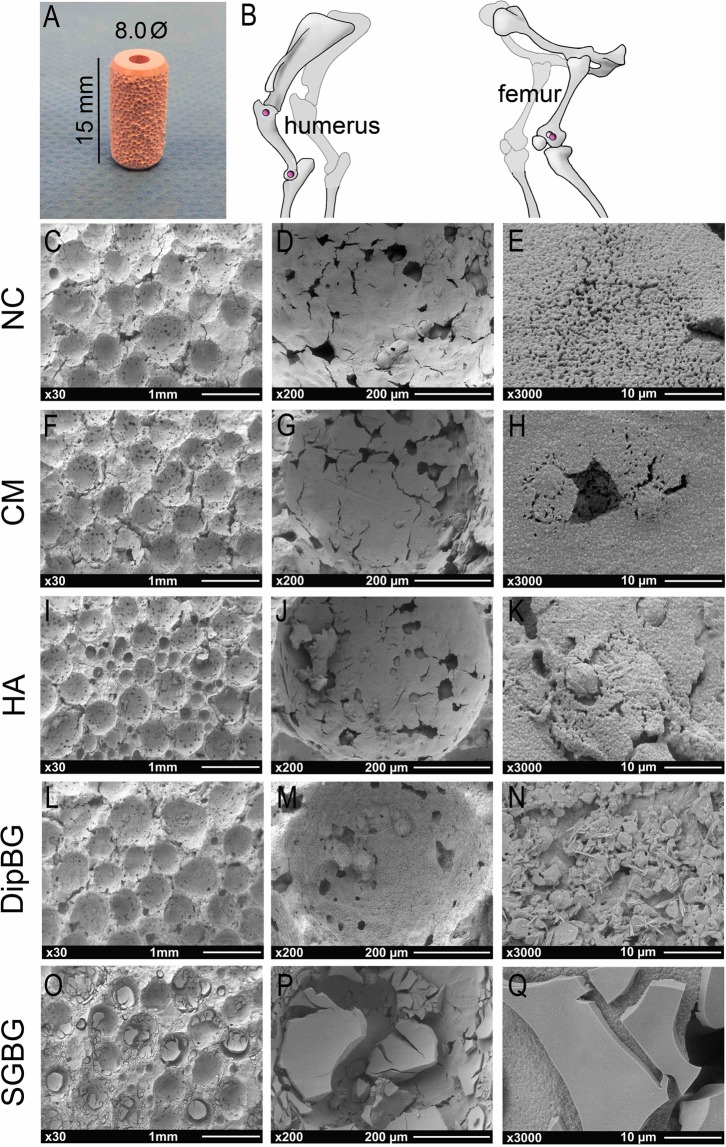
Implant design, implantation sites and characterization of bioactive coatings via SEM. SEM images of coated surface in magnifications x30, x200, and x3000. Textured cylindrical ceramic implant (**A**). Used implantation sites in cancellous bone of the left proximal and distal humerus and both left femoral condyles (**B**); dark pink = lateral approach; light pink = medial approach). SEM images of NC and coating groups (**C**–**Q**).

A textured ZTA ceramic substrate was used for the implant body (CeramTec GmbH, Lauf, Germany), consisting of 75 wt% aluminum oxide (a-Al_2_O_3_), 23 wt% zirconium oxide (tetragonally stabilized ZrO_2_), 1 wt% yttrium oxide (Y_2_O_3_), 0.3 wt% chromium oxide (Cr_2_O_3_), and 0.7 wt% strontium oxide (SrO). A standard cylindrical geometry with a height of 15 mm and a mean diameter of 8 mm was chosen for the test implant (Fig. 1). For a k-wire guided implantation, and to enable standardized push out tests, all implant bodies were equipped with a central hole of 2.4 mm in diameter and a chamfer at both implant ends for a standardized implantation into the drilled bone defects. In these standardized ceramic test implants, the implant surface was textured with a porosity of approximately 47% and a pore size of 200–500 µm to enhance and improve the contact area to the surrounding bone (Fig. 1A). The surface texture was realized as described previously^12^. In short, a ceramic substrate was spray-coated with a slurry including spherical organic pore formers. During the subsequent sintering step the pore forming agents were removed, leaving spherical pores in the surface. Mechanically unstable parts were removed using a blasting process. Sixteen single-packaged and gamma sterilized (25kGy) implants were tested *in vivo* per group. Implants were either non-coated (NC) or provided with one of four different bioactive coatings. The biomechanical push-out test was performed on eight implants of each group, while the other eight implants were used for histological analysis. Uncoated implants served as control.

### Bioactive coatings

The different surface coatings were designed to mimicking the natural bone surface, but contained distinct differences in coating technology, thickness and properties.

1. A layer of covalently bound multi phosphonate molecules (SurfLink^®^, Nano Bridging Molecules SA, Gland, Switzerland) was applied on the implants in the CM group. As this modification mimics hydroxyapatite, one of the main constituents of bone, an increased adhesion of osteoblasts and improved direct bony integration was expected. The coating (1 nm thickness) is stable against chemical and enzymatic hydrolysis.
2. A thin nanocrystalline hydroxyapatite coating (20 nm) (HA^*nano*^ from Promimic AB, Mölndal, Sweden) was applied to implants in the HA group^13^. Prior to coating the implants were ultrasonically cleaned in water and isopropanol. In short, the surface was realized via a wet-chemical based process comprising three main steps. In the first step, the textured implant was fully coated by a drop-wise addition of the coating solution, a dispersion of nano-sized HA, onto the substrate, thereby ensuring full coverage of the implant. In a second step excess solution was removed by spinning. Finally, a short heat treatment removed the coating solution and lead to a nanometer thin, fully crystalline hydroxyapatite surface. As HA consists of calcium and phosphate, the major components of bone tissue, this coating is osteoconductive and highly biocompatible. The dissolution of the coating works as a stimulus for new bone formation and improves biological integration.
3. In the DipBG group BG of 45S5 composition (45 wt% SiO_2_, 24.5 wt% CaO, 24.5 wt% Na_2_O, and 6 wt% P_2_O_5_) was used as a coating. The glass powder of average particle size of 2.0 μm was processed to a slurry, dip-coated onto the ZTA ceramic, and sintered at 1000 °C for 30 min. The final coating had a thickness of at least 2 μm. The bioactive properties of 45S5 glass result from the ability of the material to form a bone-like mineral layer of hydroxyl carbonate apatite (HCA) on its surface with structural and chemical similarities to naturally occurring hydroxyapatite in bone. A tight connection between the BG and bone is established by the recruitment of osteoblasts and collagen fiber integration in the HCA layer.
4. In the SGBG group the BG coating consisted of 70 mol% SiO_2_ and 30 mol% CaO, and was applied on the ceramic implants by a sol-gel process including a heat treatment at 700 °C^14^. The coating had a thickness of approximately 5 μm.

### Scanning electron microscopy of samples

#### Surface characterisation

**Figure 2:**
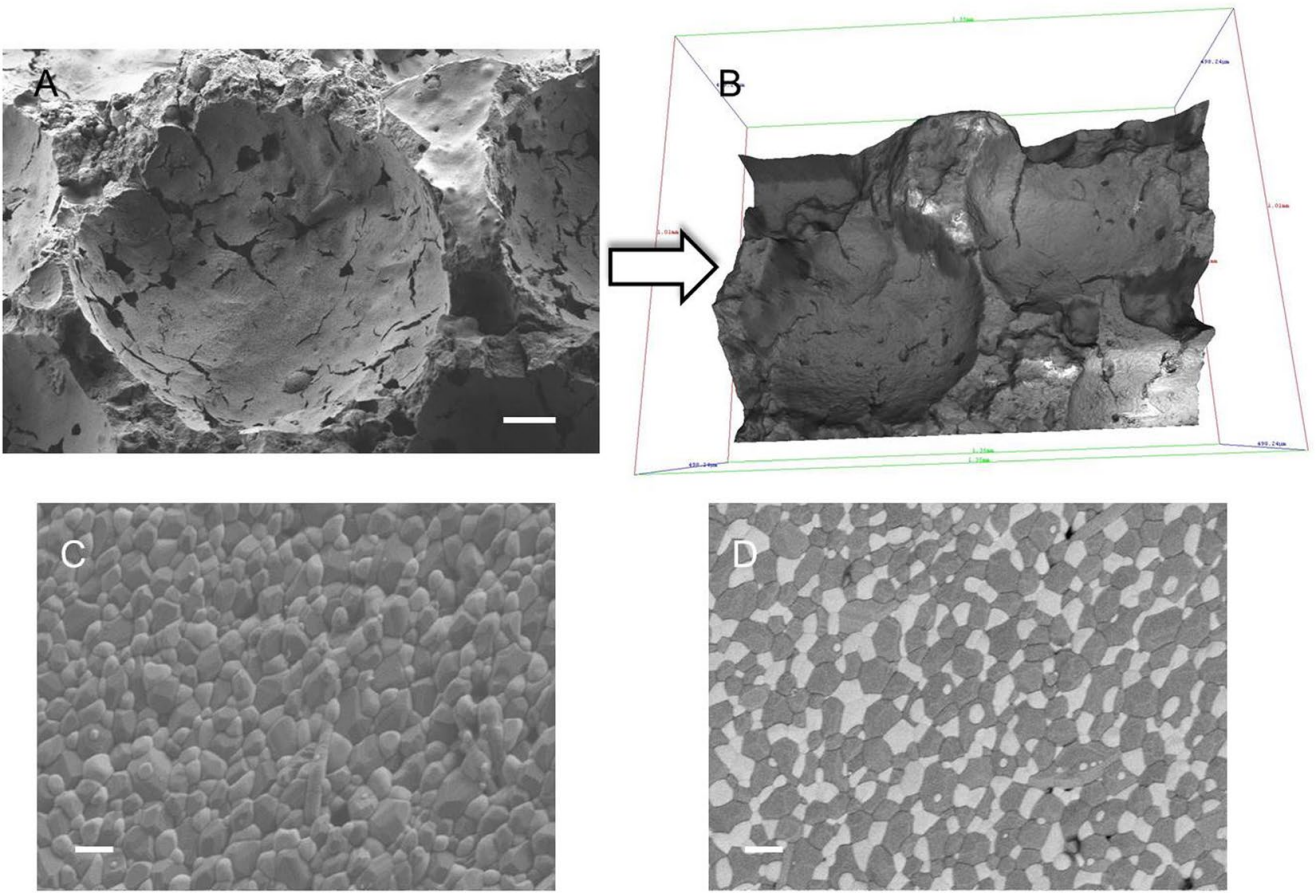
Characterization of the non-coated ZTA ceramic implant via SEM imaging in different modi. The pores of the textured surface structure are shown in low magnification (**A**), scale bar 100 µm, X 250) and visualized by a 3D model of the pores (**B**). Secondary (**C**) and backscattered (**D**) detectors applied on non-coated sample in higher magnification (scale bar: 1 µm, X 20.000).

**Figure 3:**
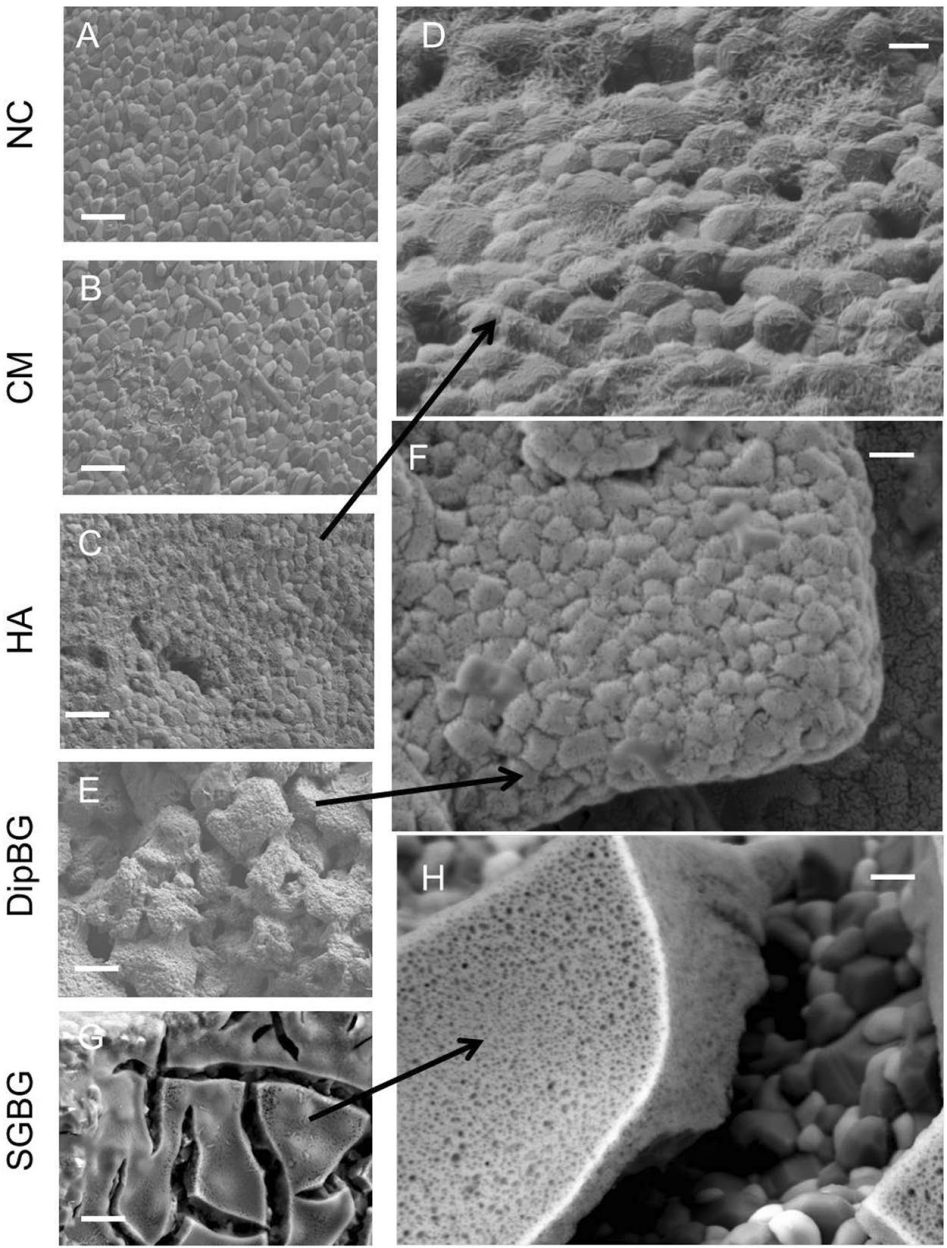
Bioactive surface coating in higher magnifications using SEM. Coating of textured implants modified the textured surface depending on the coating thickness (**A**–**H**; **A**–**C**, **E**–**G**; scale bar: 2 µm, x20.000; D,F,G; scale bar: 500 nm, x40.000).

To evaluate the surface topography and structure of coated and non-coated implants scanning electron microscopy (SEM) analysis was performed. One representative implant of each type that was not used for implantation was examined at a randomly chosen location and at different magnifications (x30, x200, x3,000, x20,000, x40,000). Images with up to x3,000 magnification were performed with Zeiss DSM 982 Gemini (Carl Zeiss Microscopy GmbH, Germany) with an acceleration voltage of 5 kV at 12 mm working distance (Fig. 1C–Q). The implants were sputtered with a layer of gold to eliminate any charging effect and to improve the contrast. For images with higher magnification field-emission SEM analysis with model Auriga (Carl Zeiss Microscopy GmbH, Germany) was used without implant sputtering (Fig. 2A–H). A primary beam energy of 1.5–4 keV as well as secondary and backscattered detectors were used. For determination of the implant structure, a 3D model was generated from three SEM stereo images by the use of special software (MeX 5.0; Alicona Imaging, Austria) (Fig. 3A–D).

### Chemical composition

X-ray photoelectron spectroscopy (XPS) was used to determine the surface chemistry of CM coated samples by an Axis Ultra spectrometer (Kratos, Manchester, U.K.) equipped with a concentric hemispherical analyser and using of a monochromatised aluminium anode X-ray source (Al KR 1,2 1486.6 eV, full width at half maximum 0.85 eV, 15 kV, 150 W). Spectra were referenced in the C1s spectrum to C-H/C-C at 285.0 eV. Spectra were evaluated using CasaXPS (version 2.3.14) and were decomposed by assuming a Gaussian/Lorentzian (70%/ 30%) peak shape. Survey spectra were recorded between 0–1200 eV binding energy. High resolution spectra were recorded for the following elements, at their respective regions: Zr3d, Al2p, Y3d, 01 s, C1s, and P2p. Any elemental concentration below 0.3 atomic % is at the detection limit.

### Animals

**Figure 4:**
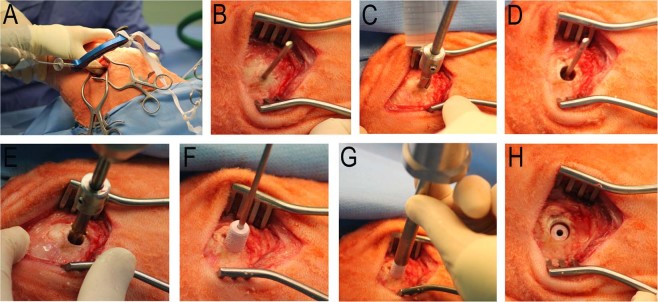
Surgical procedure. For simultaneous approach to the medial and lateral femoral condyles a fluoroscope and a k-wire drill guide were used to ensure exact horizontal placement of both implants (**A**). Over the k-wire a cannulated headed reamer (7 mm diameter) set up the drill hole (**B**–**D**). The drill hole was enlarged by the use of a custom-made headed reamer, with a 0.1 mm smaller diameter than the specific implant for a press-fit fixation (**E**). The ceramic implant was positioned central in the drill hole over the k-wire with gentile impaction on the pusher (**F**–**H**).

All animal experiments were approved by local authorities (approval number G0340/13, Landesamt für Gesundheit und Soziales, Berlin, Germany) and conducted in accordance with the EU Directive 2010/63/EU, and implemented national legislation and regulation for the care and use of laboratory animals. 20 mature merino-mix sheep ( > 2.5 years old) underwent surgery for implant testing. Four implants per animal were fixed in the cancellous bone of the epimetaphyseal region of the left proximal and distal humerus as well as both femoral condyles (Fig. 1B). These locations are adapted from previously published drill hole defect models in sheep and are commonly used for the investigation of biomaterials in cancellous bone^15–17^. Using a fluoroscope and a drill guide for a k-wire (Arthrex® RetroConstruction™ Drill Guide, Munich, Germany), the exact placement of both implants proximal to the fossa intercondylaris and horizontally to the condyle ends was ensured (Fig. 4A,B). Using the guide wire, and a cannulated, headed reamer (Arthrex, Munich, Germany), a 7 mm diameter drill hole was set up to a depth of 15 mm under constant irrigation with saline solution. A custom-made drill stop ensured the exact depth (Fig. 4C,D). Using a custom-made headed reamer, the drill hole was created 0.1 mm smaller than the implant for a standardized press-fit implantation (Fig. 4D). The drill hole was carefully flushed with saline solution to eliminate bone debris and dried with sterile compresses before placing the ceramic implant with its central hole over the k-wire. With a custom-made pusher and slight impaction, the sample was positioned just below the bone surface (Fig. 4E–H). A total of 80 ceramic implants with four different coatings and the uncoated control were distributed by a randomized completed block design to 20 animals (mean weight 76 kg; standard deviation ± 7 kg). This statistical procedure led to a homogenous distribution of test samples to the 20 animals and bone locations, lowering the influence of implant location both within and between individual test subjects.

### Anesthesia procedure

An earlier established anesthesia procedure was performed^16^.

### Surgical procedure and animal care

3 g amoxicillin (Unacid^®^, Pfizer Pharma GmbH, Karlsruhe, Germany) and 500 mg metronidazol (Metronidazol infusion solution, Fresenius Kabi GmbH, Bad Homburg, Germany) were given intravenously as a single-shot antibiotic. 8,000 IE Dihydrostreptomycinsulfat, 4.8 mg Benzylpenicillin- Procain, and 3 mg Benzylpenicillin-Benzathin (Veracin®, Albrecht GmbH, Aulendorf, Germany) per kg body weight were given subcutaneously for 8 days. Sheep received a 75 μg/h fentanyl patch (Durogesic^®^ 75 μg/h, Jannsen-Cilag GmbH, Neuss, Germany), which was exchanged three days post surgery, and a dose of 2.2 mg flunixin-meglumin (Finadyne®, Intervet Deutschland GmbH, Unterschleißheim, Germany) per kg body mass for seven days was used for analgesia.

After an implantation period of 12 weeks, all animals were sacrificed by intravenous application of an over dosage of thiopental sodium (Trapanal®, Altana Pharma GmbH, Konstanz, Germany) followed by potassium chloride and explantation of humeri and femora for biomechanical and histological analysis.

### Biomechanical uniaxial push-out test

#### Sample preparation

Humeri and femora of ten animals were used for biomechanical uniaxial push-out testing. The samples were cleaned from surrounding muscles, tendons and soft tissue until the implant located below the bone surface was visible. With a custom-made sawing device (Centrum Wissenschaftliche Werkstätten, Charité - Universitätsmedizin Berlin, Germany) a straight cut (Makrotrennschleifsystem, Exakt, Norderstedt, Germany) below and perpendicular to the implant was performed. With a second custom-made milling device, the implant end was cleaned below from bone in a diameter of 9 mm. During preparation, all samples were cooled and kept moist with saline solution.

To allow analyses of baseline positioning and baseline bone contact, a cadaver test was performed identically to the surgical procedure described above. At sacrifice, the contralateral limbs of the eight uncoated control implants were used for quantitative analyses of push-out forces at week 0 (without healing time).

#### Test setup

**Figure 5:**
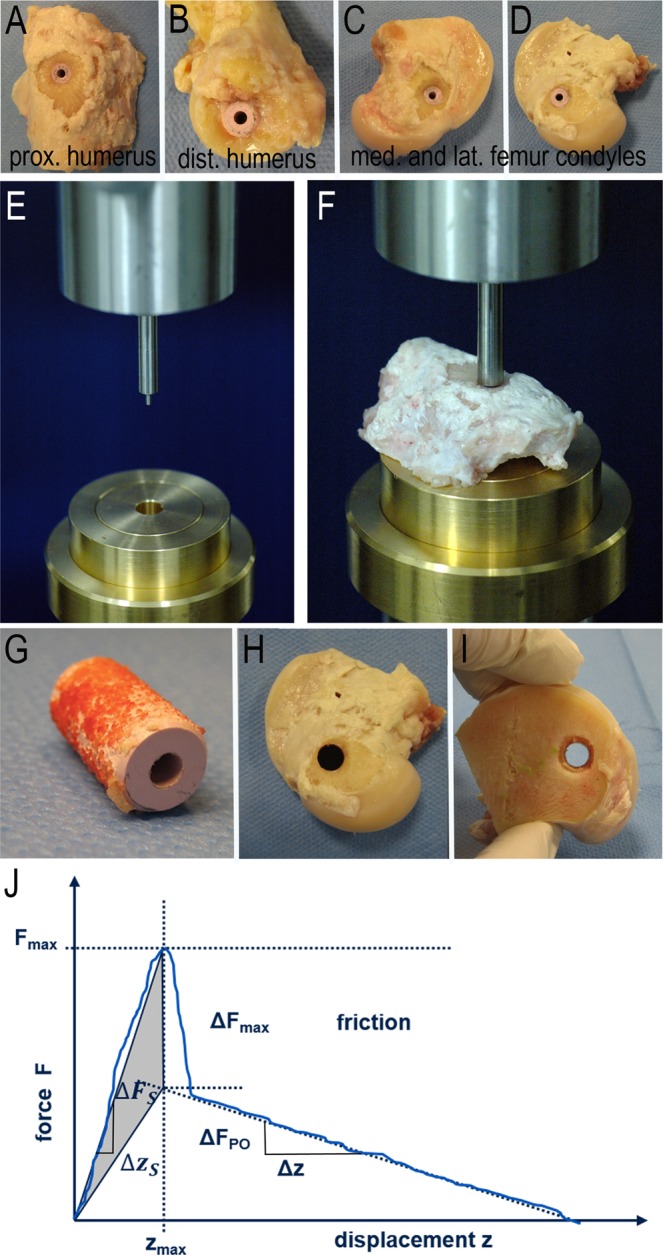
Biomechanical push-out test procedure. Explanted samples, were freed from surrounding soft tissue and bone was trimmed for vertical placement under the punch (**A**–**D**). The punch with central mandrel (Ø 2.2 mm) was fixed to the upper traverse (Zwick/Roell Z010, Ulm, Germany) and positioned in the central hole of the implant. A brass alloy ring was attached to the lower traverse (**E**). The bone sample was placed over the central hole of the ring with the flat surface down (**F**). Push-out force and implant displacement were continuously acquired during the test until the ceramic implant and bone were out of contact (**G**–**J**; graph adapted from^49^).

With the uniaxial push-out test, the biomechanical quality of osseous implant integration was analyzed (Fig. 5). The prepared ceramic implants (Fig. 5A–D) were pushed out of the cancellous bone, while the required force and implant displacement were recorded. In a Zwick testing machine (Zwick/Roell Z010, Ulm, Germany) a punch with a central mandrel (Ø 2.2 mm) was fixed in the upper traverse, while a brass alloy ring was attached to the lower traverse (Fig. 5E). The flat surface of the bone sample was positioned above the central hole of the ring, while the mandrel of the punch was positioned in the central hole of the implant (Fig. 5F). With an initial testing speed of 5 mm/min the punch was moved towards the sample until a reaction force of 3 N was reached. Then the testing speed was increased to 10 mm/min. The push-out force [N] and the implant displacement [mm] were recorded (TestXpert II, Zwick, Ulm, Germany) until the ceramic implant was completely pushed out of the bone (Fig. 5G–J). Abort criterions of the test were defined as a displacement of 18 mm or a force of 8500 N. Three samples (one of CM, SGBG and DipBG group) needed to be excluded from the analysis as the punch was bent and wedged in the sample leading to a test failure (n = 8 NC, n = 7 CM; n = 8 HA; n = 7 DipBG; n = 7 SGBG).

#### Parameters

All parameters were calculated from force - displacement data using a custom analysis script (MATLAB R2009b, The Mathworks Inc. Natick, USA). The force required to initiate breakage at the bone implant interface was defined as maximum push-out force [Fmax in N] (maximum of the load-displacement curve, Fig. 5J). The adhesive shear strength was calculated as maximum push-out force divided by the outer surface of the implant [$\tau {\rm{A}}{\rm{S}}=\frac{Fmax}{hImp\ast \pi \ast dImp}$; in MPa]. The shear stress by friction was determined by linear regression analysis of the descending part of the curve [$\tau {\rm{S}}{\rm{F}}=\frac{\Delta FPo}{\Delta z}\frac{1\,}{\pi \ast dImp}$; in MPa]. To compute the energy release rate, the released energy at the point of implant failure was divided by the area of the released outer surface of the implant [$G=\frac{\Delta {\rm{F}}{\rm{m}}{\rm{a}}{\rm{x}}\ast {\rm{z}}{\rm{m}}{\rm{a}}{\rm{x}}}{2\ast {\rm{M}}{\rm{I}}{\rm{m}}{\rm{p}}}$; in J/m^2^]. The implant bone interfacial stiffness was determined by linear regression analysis of the ascending part of the curve [$S=\frac{\Delta \mathrm{Fs}}{\Delta \mathrm{Zs}}$; in N/mm]. The energy to failure was determined by the area under the ascending part of the curve [$EPo={\int }_{0}^{Zmax}Fpo(z)dz\,$; in J] and indicates the amount of work that must be applied to the system to reach the breakage point.

### Histology

#### Sample preparation

Samples were harvested 12 weeks after surgery for undecalcified bone histology of thin sections, which were prepared using a sawing and grinding technique^18^. The samples were fixed for five days in 4% formaldehyde solution and embedded in Polymethylmethacrylate (Technovit 9100new, Heraeus Kulzer-GmbH, Hanau, Germany). The thin section was ground to approximately 100 µm thickness and polished. Surface staining was performed with Giemsa; coloring mineralized bone matrix in pink, osteoid in blue, and cartilage in purple.

#### Evaluation

**Figure 6:**
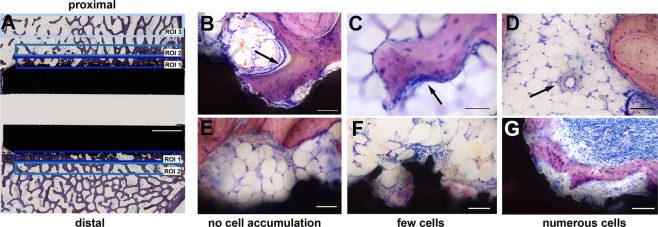
Histomorphometrical and –morphological analysis. Three Regions of Interest (ROI 1–3) were defined in each sample (**A**; ROI 1 = dark blue box; ROI 2 = blue box; ROI 3 = light blue box; white scale bar = 2500 µm). The trabecular bone surface covered with a layer of osteoblasts and osteoid (**B**; arrow) was measured. Osteoclasts count (C; arrow) on the trabecular bone surface estimated for or the osteoclast density. Vessel count (**D**; arrow) for the estimation of the vessel density. Accumulation of inflammatory cells surrounding the implant (ceramic surface = black area) was scored according to the criteria: no accumulation of cells, few cells, or numerous cells (**E**–**G**; scale bar = 100 µm).

For the histomorphometrical analysis, the sections (Fig. 6A) were digitized using a 5.0x microscope lens (Axioskop 40, Carl Zeiss MicroImaging GmbH, Göttingen, Germany) and AxioVision software (AxioVision Rel. 4.8 Carl Zeiss Imaging Solutions GmbH, 2005, Göttingen, Germany). Three Regions of Interest (ROIs) were defined using ImageJ (version 1.50a, Rasband, W.S., U. S. National Institutes of Health, Bethesda, Maryland, USA). The first area around the proximal and distal implant surface was defined as ROI 1 starting from the deepest point of the textured surface with a height of 1 mm and a length of 14 mm corresponding to the length of the textured surface on the implant (Fig. 6A; dark blue box). ROI 2 was defined as the second area adjacent to the proximal and distal implant surface with a height of 1 mm and a length of 14 mm (Fig. 6A; blue box). ROI 3 had a defined size of 2 mm in height and 15 mm in length and was located in the native surrounding trabecular bone which was not affected from the implantation. ROI 3 was used to measure the normal mineralized bone area of the trabecular bone (2D bone density; Fig. 6A; light blue box). Within these ROIs the mineralized bone tissue and connective tissue was measured in mm² and given in percentage of the corresponding total area of the ROI (in %). The direct bone implant contact (BIC) was measured on the proximal and distal side of the implant and given in relation to the total surface of the textured implant (in %).

The trabecular surface covered with a layer of osteoblasts and osteoid (Fig. 6B), as a sign of active direct bone formation, was measured and given in relation to the total trabecular surface in the proximal and distal ROI 1 (in %). To allow quantitative analyses, all sections were digitized using a 10.0x microscope lens.

The osteoclast density of the bone area was determined under a 20.0x microscope lens in the proximal and distal ROI 1. As no osteoclast-specific staining can be performed on thin sections, the criterion for inclusion of cells to the count were two or more nuclei, a typical morphology, and the location on the bone surface (Fig. 6C). The amount of osteoclasts was related to the mineralized bone tissue area (n/mm²) to obtain the osteoclast density.

The vessel density in the connective tissue in proximal and distal ROI 1 was determined using the 20.0x microscope lens. The counts were related to the whole connective tissue area of ROI 1 (n/mm²). As no specific vessel staining can be performed on thin sections, only larger vessels starting from approximately 10–20 µm with a defined lumen and a detectable vascular wall were counted (Fig. 6D).

Qualitatively, host tissue response to the implant was assessed for signs of inflammation and foreign body reaction. The accumulation of inflammatory cells at the bone-implant interface was classified into three groups: no accumulation of cells, few cells, and numerous cells (Fig. 6E–G).

### Statistics

Statistical analyses of data were performed using SPSS (SPSS® 20.0 GmbH Software, Munich, Germany). A p-value of < 0.05 was considered as significant. Normal distribution of data could not be confirmed. A univariate ANOVA with post hoc test Dunnett’s T3 was performed for multiple comparisons between the four groups and the control group (biomechanical and histomorphometrical data). Comparison of histomorphometrical data (ROI 1–3) within a group was performed using the Wilcoxon signed rank test. Data are presented in box plots showing the median, 25^th^ and 75^th^ percentile, and min–max (whiskers).

## Results

### Scanning electron microscopy of coated implants

The SEM images illustrate the surface morphology of the different groups. CM and HA coating did not affect the textured surface structure on a micrometer scale in comparison to a NC sample (Fig. 1C–K), while the two thicker BG coatings modified the textured surface by partial filling of the ceramic pores (Fig. 1L–Q).

The hemispherical surface structures, which were ranging between 200–500 µm in diameter, were clearly detectable and the effect of the surface engineering was visualized by a 3D reconstruction (Fig. 2A,B). By applying high magnification and backscattered (Fig. 2D) in comparison to secondary (Fig. 3C) detectors, the ceramic surface morphology of the ZTA ceramic implant was evaluated. Al_2_O_3_ (dark) and ZrO_2_ (bright) ceramic grains are clearly distinguishable (confirmed by EDX analysis, data not shown). The surface morphology of the different coatings was analysed only by the secondary detector (Fig. 3). While no difference in the surface morphology between control and CM were analysed (Fig. 3A,B), strong varieties of the surfaces were achieved with the other coatings (Fig. 3C–H). As CM can be seen as a kind of bio-functionalisation on the nanometer scale it is obvious that this treatment will not change the surface morphology. The HA coatings resulted in an increase of nano roughness, as HA^nano^ needles are coated on the surface (Fig. 3C,D). On the DipBG coating surfaces, the used BG particles are visible and an increase of surface roughness is observable even in the sub-micrometer scale (Fig. 3E,F). After drying of the SGBG coatings, cracks and island structures occurred on the ceramic samples (Fig. 3G,H). This is a typical effect usually happening during the drying of thick sol-gel layers caused by mechanical stress inside the coating^19^ and indicates that these coatings were not ideal in terms of their thickness and topographical structure.

### Chemical composition

Determination of the chemical composition of the CM biomaterial surface was performed by means of XPS measurements (Table 1). The presence of the phosphorus peak proved a successful SurfLink^®^ treatment.

**Table 1:** CM coating - atomic concentration of elements.

element	mean value in % (n = 6)	standard deviation in %
O 1 s	45.4	6.3
C 1 s	24.9	8.1
Zr 3d	3.8	0.4
Y 3d	0.2	0.0
Al 2p	23.6	1.5
P 2p	1.7	0.2
Si 2 s	0.4	0.2

### Clinical examination of animals and *in vivo* results

All animals recovered from surgery and anesthesia without complications. Sheep were kept in groups without restriction to movement and showed a full weight bearing on the operated limbs immediately after surgery. A slight to moderate lameness of the operated legs was detectable the first days after surgery. This was treated according to severity with flunixin-meglumin, fentanyl patch, or buprenorphine (Temgesic, Essex Pharma GmbH, Munich, Germany) until full recovery. The general health status was never affected.

### Macroscopic analyses and biomechanical push-out test

**Figure 7:**
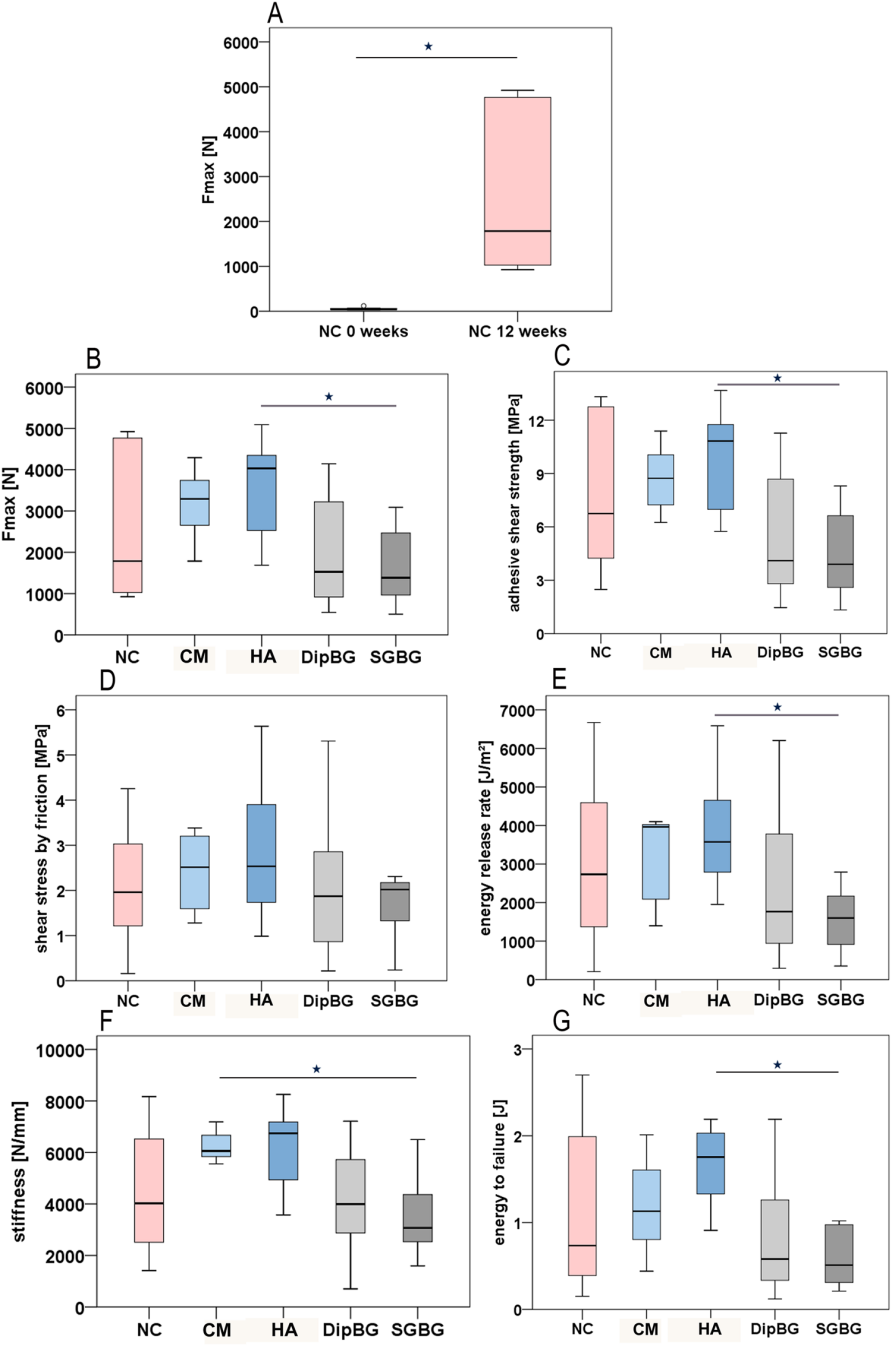
Biomechanical push-out test results. The NC group showed a significant increase of Fmax from week 0 to 12 (**A**; p = 0.006). Comparing all groups after 12 weeks, HA group achieved a significantly higher Fmax compared to the SGBG group (**C**; p = 0.046). The adhesive shear strength was significantly higher for the HA group compared to SGBG coating (**C**; p = 0.025). The shear stress by friction showed no differences between all groups (**D**). The HA coating had a significantly higher energy release rate compared to the SGBG coating p = 0.032 (**E**; p = 0.032). CM coating had a significantly higher interfacial stiffness compared to the SGBG coating (**F**; p = 0.038). The energy to implant failure was significantly higher in the HA group compared to the SGBG group (**G**; p = 0.002).

Macroscopically, remnants of attached bone were visible on the implant surface after push-out testing in all groups. In two samples of the CM and HA group, and one sample of the control and DipBG group, a thicker bone layer still covered larger parts of the implant after the test. The comparison of NC implants at week 0 (cadaver test) to 12 weeks showed a statistically significant higher maximum push-out force (Fmax; p = 0.006; Fig. 7A). After 12 weeks healing time, at least a seven-fold increase in Fmax was detectable. However, the NC group showed a high variability in Fmax between 1000 and 5000 N. Comparing the four coated groups, a clustering of test results was detectable; while CM and HA coating showed comparable results with a tendency to increase Fmax, both BG coatings showed similar results, with the tendency to decrease Fmax. A statistically significant difference was detectable for HA and the SGBG coating in Fmax. (HA: mean 3573.85 ± 1119.91 N; SGBG: mean 1691.57 ± 986.76 N; p = 0.046; Fig. 7B) and the adhesive shear strength (HA: mean 9.82 ± 2.89 MPA; SGBG: mean 4.57 ± 2.65 MPA; p = 0.025; Fig. 7C). The shear stress by friction showed no statistically significant difference between the groups (Fig. 7D). The comparison of the energy release rate showed a significantly higher rate in the HA coating in comparison to the SGBG coating (HA: mean 3821.95 ± 1474.13 J/mm^2^; SGBG: mean 1558.47 ± 923.47 J/mm^2^; p = 0.032; Fig. 7E). The implant-bone interfacial stiffness showed a significant increase by CM coating in comparison to SGBG coating (CM: mean 6258.06 ± 603.80 N/mm; SGBG: mean 3565.57 ± 1705.31 n/mm; p = 0.038; Fig. 7F). The energy to implant failure was significantly higher in the HA group compared to the SGBG group (HA: mean 1.67 ± 0.45 J; SGBG: mean 0.62 ± 0.36 J; p = 0.002; Fig. 7G).

### Descriptive histology and histomorphometry

**Figure 8:**
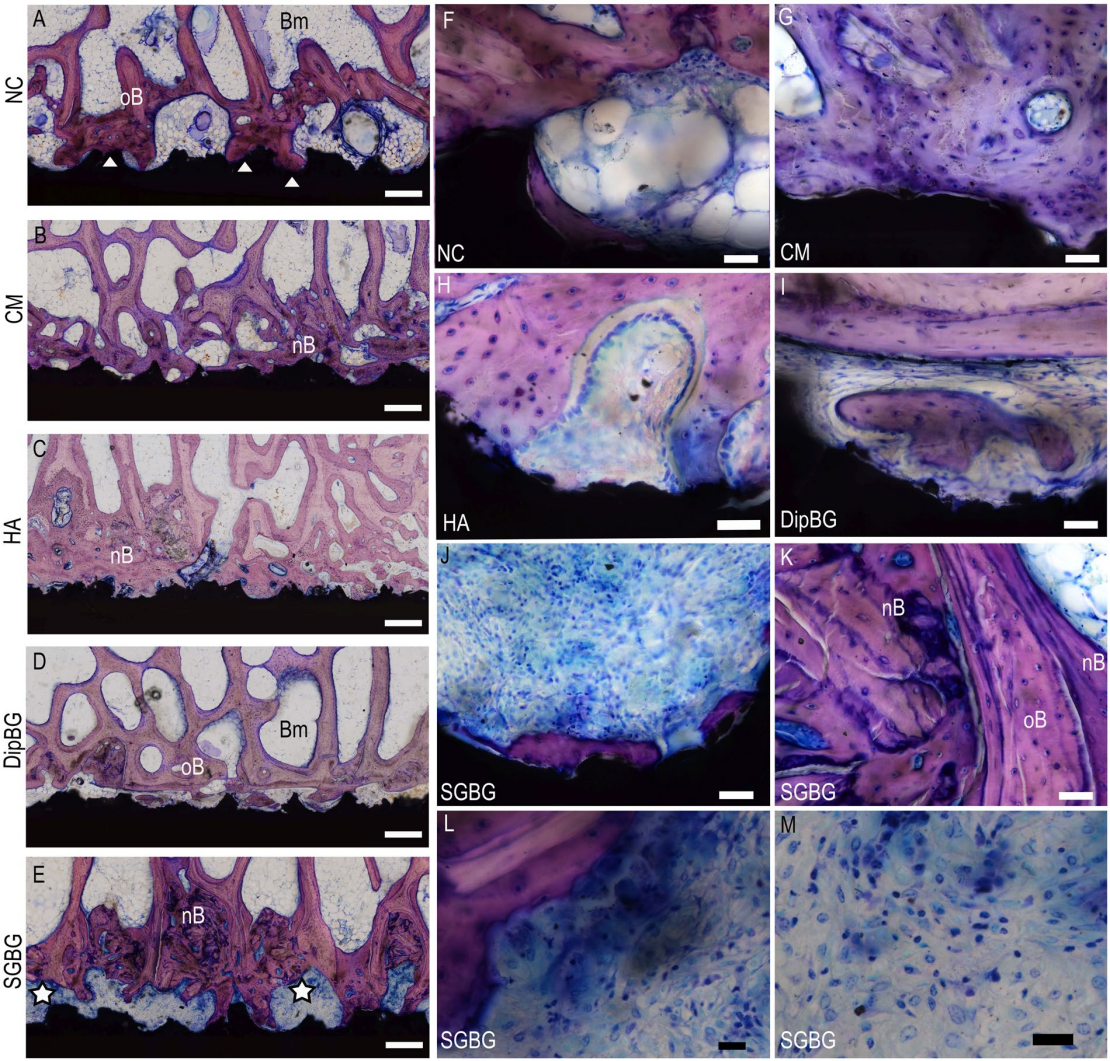
Histomorphological evaluation. One sample of each group is shown exemplarily (**A**–**E**; scale bar = 500 µm; F-M magnifications of **A**–**E**), with the textured ZTA ceramic surface in black, while surrounding trabecular bone was stained in pink (Giemsa). Direct bone-implant contact exemplarily marked with a white triangle in the sample of the NC group (**A**). Fatty bone marrow fills the areas between the trabeculae (Bm = Bone marrow). Old host bone is stained in lighter pink (oB = old Bone), while newly formed bone is stained darker (nB = new Bone). The connective tissue surrounding the SGBG group implants was filled with a layer of blue stained cells consisting of lymphocytes, plasma cells, and macrophages as a foreign body reaction to the coated implant (**E**; stars; **J**, **L**, **M** as magnifications).

The ceramic implants showed varying bony integration (Fig. 8). While most samples in NC, CM, and HA groups showed a dense bony implant integration (Fig. 8A–C,F–H), most samples in BG groups showed a less dense bony integration (Fig. 8D–E,I,J). The bone formation process was direct via osteoblasts in all groups, no indirect bone formation was detectible. Surface osteoblasts formed new bone appositionally on already existing trabeculae, apparently by large active osteoblasts and osteoid seams (Fig. 8H). Furthermore, woven bone was formed between the trabeculae leading to a higher bone density around the implant (Fig. 8B–E,K). New bone attaching to the implant surface (Fig. 8A; triangles) was connected to old bone trabeculae, which were distinguishable in most cases by a slighter pink color (Fig. 8K). However, small areas with bone formation directly on the implant surface were detectable throughout all groups (Fig. 8J). Areas adjacent to the implant without direct bone bonding were filled with fatty bone marrow in the NC, CM, HA, and most DipBG samples, comparable to native trabecular bone areas (Fig. 8A,F). However, all samples in the SGBG group showed a layer of loose connective tissue with different levels of accumulated inflammatory cells at the implant tissue interface (Fig. 8E; white stars). The accumulated cells were lymphocytes, plasma cells, and macrophages, forming a dense layer of numerous cells in half of the SGBG samples (Fig. 8J,L,M as magnifications of E). In the other half, samples showed fewer cells and a less dense infiltration. Despite the present foreign body reaction, active direct bone formation was detectable around the implant in the SGBG group (Fig. 8J,K). Only in three samples of DipBG coating a few inflammatory cells surrounded the implant in clusters. One sample of NC showed the accumulation of a few inflammatory cells around the implant. No sample of the CM and HA group showed an accumulation of inflammatory cells.

**Figure 9:**
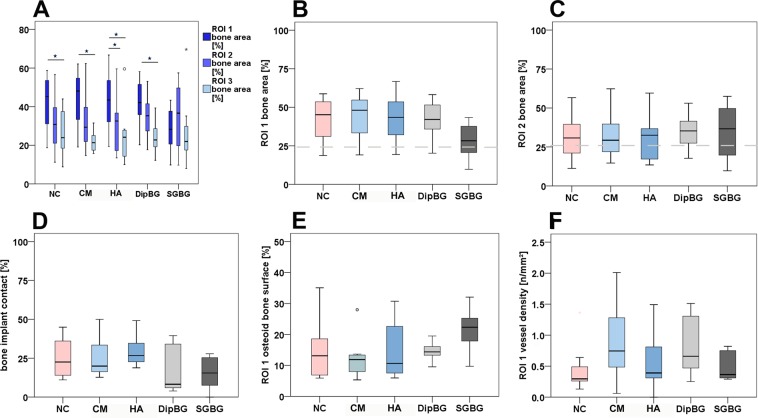
Histomorphometrical and –morphological results. Comparison of bone area in ROI 1 compared to ROI 2 or 3 within a group (**A**; Wilcoxon signed rank test; ROI 1 vs. ROI 2: HA p = 0.012; ROI 1 vs. ROI 3 NC p = 0.017; CM p = 0.012; HA p = 0.012; DipBG p = 0.017). Comparison of bone area in ROI 1 or ROI 2 showed no difference between the five groups (**C**; dashed grey base line at 24,96% = mean bone density of native cancellous bone). Comparison of direct bone implant contact (**D**), osteoid layer on bone surface (**E**), and vessel density (**F**) showed no difference between the groups.

The histomorphometrical evaluation confirmed the histomorphological findings. In all groups, bone density (2D) increased adjacent to the implant (ROI 1) compared to native trabecular bone (ROI 3) within a group, except in SGBG group (Fig. 9A; Wilcoxon signed rank test: NC p = 0.017; CM p = 0.012; HA p = 0.012; DipBG p = 0.017). Additionally, in the HA group an increase in bone area was also detectible comparing ROI 1 with ROI 2 (Fig. 9A; Wilcoxon signed rank test: HA p = 0.012). However, comparing the bone area in ROI 1 between the five different groups, no statistically significant difference was detected (Fig. 9B). The SGBG group showed the lowest 2D bone density in ROI 1. The comparison of the bone area in ROI 2 between the five groups showed no statistically significant difference; the SGBG group however, showed higher values of bone area in this ROI (Fig. 9C).

The bone implant contact (BIC) indicated no statistically significant difference between the coating groups and the uncoated control group. The highest median value was measured in the HA group (Fig. 9D).

All groups showed active bone formation 12 weeks after surgery in the proximal and distal ROI 1, reflected in the percentage of osteoid layer covering the trabecular bone surface (Fig. 9E). No statistically significant difference could be detected between the groups.

The osteoclast density in the proximal and distal ROI 1 was almost zero in all groups. No events of active osteolysis around the implant were detectable 12 weeks after surgery in any group.

The vessel density of the connective tissue in the proximal and distal ROI 1 showed no statistically significant difference between the groups. Highest values were detectable in the CM group (Fig. 9F).

## Discussion

To improve the bony integration of mechanically loaded ZTA ceramic implants into cancellous bone, the combination with four different bioactive coatings was investigated in the present study. The chosen bioactive coatings should enable direct bone bonding, by mimicking the natural bone surface. The aim of the present study was to analyze the biological integration of textured ZTA ceramic implants with bioactive coating in a, mechanically loaded implantation site of a relevant large animal model. Osseointegration of standardized cylindrical ZTA ceramic implants in the epimetaphyseal region of cancellous sheep bone were analyzed after 12 weeks by a biomechanical push-out test and histological evaluation.

The sheep model was chosen to facilitate a comparison to human bone turnover and mineral apposition rates^20,21^. The drill hole defect model in the epimetaphyseal bone region is widely accepted as a mechanically pre-loaded bone defect without massive micro-movements that would overlay biological adaptation and regeneration processes^15–17^. For the specific implants of the present study, the implantation sites of both femoral condyles and an observation time of 12 weeks was chosen for an early investigation of the healing response^20^. It can be assumed that the chosen location and limb loading situation would be roughly comparable to weight bearing in the weeks after implantation of a non-cemented prosthesis^22^.

The results of the present study showed a stable mechanical integration of the uncoated ceramic implants in the biomechanical push-out test after 12 weeks. The maximum push-out force increased at least seven-fold from week 0 to 12 weeks post implantation. With respect to the four different coatings, a clustering of CM and HA group results and both BG group results were remarkable. While the thin coatings of CM and HA stabilized the osseointegration of ceramic implants compared to the control group, which showed a wide variability in all biomechanical parameters, this effect was missing in both BG groups, characterized by relatively thick coatings. A statistically significant increased maximum push-out force, adhesive shear strength, energy release rate, energy to failure, and total energy was detected between the HA and SGBG coating. Furthermore, the implants treated with CM showed a statistically significant higher stiffness compared to the SGBG coating. The histomorphometrical and histomorphological evaluation supported the biomechanical push-out test results. While in CM, HA, and the NC group the trabecular bone density increased closer to the implant, leading to a dense bony integration in most samples, the BG groups showed a less dense to only slight direct bony integration. Additionally, the histological evaluation unraveled the potential underlying mechanism; the BG coatings showed different extents of a foreign body reaction surrounding the implant. While in the thinner, slower dissolving DipBG group only a slight reaction on some samples was detectable, a more prominent foreign body reaction was detectable in the SGBG group. A lympho-plasma cellular inflammatory reaction with macrophages was detectable in all samples that caused the delay in new bone formation and bony integration due to the sustained inflammatory environment with less new bone formation.

The coating of titanium implants with hydroxyapatite has been a focus for improving osseointegration for a long time^23–25^ and has already shown promising results in patients^26^. HA coating, composed of Ca^2+^ and PO_4_^3−^, is chemically similar to the apatite of the host’s bone^13^, and is a source of calcium and phosphate at the implant interface. The release of calcium and phosphate respectively to phosphorous from apatite surfaces is determined by the ratio of Ca/P^27^. In total hip replacement studies, an improved fixation with a decrease in the number of radiolucencies around HA coated titanium alloy femoral components has been shown^28,29^. As the chemical composition, thickness and microstructure of the HA plays a critical role for implant integration^10^, in the present study, a nano HA coating was tested for the first time on textured ceramic implants to improve osseointegration. On titanium, this coating has shown to increase the removal torque in rabbit tibiae by 20–40%, and to have a significant effect on the bone to implant contact, up to 300% higher compared to uncoated titanium^28^. The HA coating has also been tested on screw shaped polyether ether ketone implants in rabbit tibiae, leading to an increased removal torque, biocompatibility^13,29^, and bone implant contact in comparison to the uncoated control implants^13,30^. The nanometer-sized particles accelerate bone formation by facilitating adhesion of osteoblasts to the implant surface^13,31^. The chemical composition, size, and shape of deposited HA^nano^ crystals is similar to that in bone^29,30,32^, functioning as a binding site for molecules as collagen and adhesion of osteoblasts^13,31^.

In comparison to the HA coating, the treatment of the CM group consists of covalently bound molecules containing phosphate-like groups and thus this coating mimics the surface of naturally occurring HA. The CM layer is stable against chemical and enzymatic hydrolysis. The molecules provide an increased hydrophilicity and wettability of implant surfaces^33^ for enhancement of bone cell adhesion and growth^34^. On titanium, a similar coating has been investigated *in vitro* and *in vivo* with promising results. An osteoblastic cell line from rats showed increased osteoblastic activity on phosphoric acid-coated titanium disks^34^ and an increased calcium phosphate deposition^35^. *In vivo*, commercially available titanium dental implants were tested in the hip bones of sheep^33^. Removal torque for multi-phosphonate treated implants were increased compared to the uncoated control implants. Bone implant contact over time and an increased new-old bone ratio at eight weeks showed a positive tendency for multi-phosphonate treated groups^33^. In a rat study, the coating significantly increased the pull-out strength of cylindrical implants compared with control implants in the contralateral leg^36^. A pilot clinical study with the coating on titanium dental implants compared to non-coated implants showed uneventful healing and a trend for reduced peri-implant bone loss in preliminary short-term data three year post-implantation^37^.

In general, BG form a hydroxycarbonate apatite (HCA) layer on their surface by reacting with the host tissue, such layer has structural and chemical similarities to naturally occurring HA in bone. BG are reported to form a rapid, strong, and stable bond with host tissue via cation exchange, Si-OH group formation and amorphous calcium phosphate phase deposition crystallizing to hydroxycarbonate apatite, which binds to collagen^11^. The bioactive mechanisms of bone bonding and bone tissue ingrowth enhancement are assumed to result from the parallel and sequential reactions that occur at the material tissue interface as dissolution, precipitation, ion exchange reactions, and interaction with adhered cells, leading to a biologically equivalent apatite surface on the implanted material and gradual incorporation of the bioactive implant into developing bone tissue^31^. By the dissolution of ions such as Ca^2+^, PO_4_^3−^ and Si^4+^, gene expression in osteogenic cells and bone vascularization is activated, leading subsequently to the promotion of a high rate of bone formation^11,38^. In rodent models, an increased bone adhesion has been detected with BG coated zirconia implants in the epiphyses of rats compared to uncoated controls^39,40^. In addition, bioactive glass 45S5 coated porous Ti-implants have been shown to exhibit greater bone ingrowth compared to HA and uncoated implants^41^. However, in the present sheep study, the BG coating of the ceramic implants did not show the desired effect towards an improved bony integration. A foreign body reaction to the material sustained the initial inflammatory phase after implant integration and decreased bone attachment. Nevertheless, new bone formation was present adjacent to the implant as the trabecular surface was covered with osteoblast-forming osteoid surrounded by rich vascularization. The layer of connective tissue with inflammatory cells however, did not lead to a tight mechanical fixation of the implant. The varying extent of foreign body reactions might be related to the distinct characteristics of both BG coatings. The ion composition, glass particle size, and chemical composition influence the HA layer formation^42^. In the DipBG group, a thinner layer of the melt derived BG 45S5^11^ was applied by a dipping technique, while in the SGBG group a thicker layer of 70S30C was applied by a sol-gel technique^42^. 70S30C sol-gel derived materials have a mesoporous texture and high surface area, thus the rate of surface reactions is intensified, leading to faster release of ionic species during glass dissolution and a large number of nucleation sites for calcium-phosphate layer deposition compared to dense melt-derived glasses^42^, however, as these coatings underwent heat-treatment it is likely that a textured, nanostructured topography was not present. Also, it can not be excluded that especially for the thicker SGBG coating parts of the coating were chipped off during press-fit implantation. These fragments would lead to an even higher free surface area which could induce additional surface reactions.

The insertion of any implant into bone induces, in general, an acute inflammatory reaction as a response to the acute injury, typically with a limited foreign body reaction^24,43^. The acute inflammatory response usually resolves within the first week, while chronic inflammation is identified by the presence of mononuclear cells, i.e. monocytes, lymphocytes, and plasma cells confined at the implant site^43^. Macrophages are one of the first immune cells to arrive at the tissue-implant interface in the sequence of healing and inflammation processes^44^. The extravasation and migration of monocytes/macrophages to the implant site is guided by chemokines^43^. Macrophages mediate the inflammatory processes, especially chronic inflammation^44^. Activated macrophages show several heterogeneous phenotypes and secrete an array of inflammatory mediators following activation, i.e. degradative enzymes, acids, or proteins that modulate fibrosis leading to a capsule formation surrounding the implant^43,45^. Lymphocytes interact with macrophages and stimulate the formation of giant cells and enhance osteoclast differentiation as the next step in the cascade of the foreign body reaction^43^. Macrophages and activated fibroblasts disturb normal homeostatic mechanisms around orthopedic implants^45^. Activated fibroblasts secrete “osteoclastic” cytokines that suppress the osteoblast function leading to a predominance of bone resorption over osteogenesis^25,45^. If this process continues without resolution, it results in chronic inflammation and osteolysis, jeopardizing the long-term stability of the implant^24^. A comparable study in sheep investigating Ti-cylinders coated with BG in another composition (Na_2_O: 7–24wt%, K_2_O: 2–8wt%, CaO: 9–20wt%, MgO: 0.1–2wt%, Al_2_O_3_: 0.1–2wt%, SiO_2_: 46–63wt%, P_2_O_5_: 4–8wt%) implanted in the distal femoral epiphysis showed a similar mechanism as the present study^42^. In a mechanical extraction test, the mechanical failure occurred at the interface between bone and coating. The histological analysis of the interface showed fibrous tissue formation with macrophages and only minor new trabeculae formation^46^. The mode of failure was explained by the histologically verified appearance of fibrous tissue with macrophages at the bone implant interface and only slight formation of new trabeculae^46^.

In comparison to other studies using a similarly textured ceramic implant surface, the mechanical stability of implants in the present study was in the same range, however an epimetaphyseal femoral defect model in mini-pigs was used^47^. Even in comparison to the gold standard of titanium material used in another study with cylindrical implants with rough or coated surfaces in the cancellous bone of the proximal and distal femur in minipigs, the adhesive shear strength of the present ceramic implants and histological bone implant contact results were comparable^48^.

Our study is limited by the investigation of an early time point of osseous implant integration, thus the long-term effect of the coatings needs to be verified in further studies. Human implant integration studies demonstrated that maximal bone ingrowth is achieved around the 9-month postoperative period^21^.

In summary, bioactive coatings based on the biomimicry of natural bone surface can influence the cancellous osseointegration on ceramic implants, however, the quality of osteointegration can vary significantly. In CM and HA coatings, the bony integration could be enhanced towards a stronger mechanical stability, proving the hypothesis. If compared to the uncoated samples however, it is evident that the nanometer sized coatings with different chemical compositions helped to reduce the “normal” wide variability of local bonding, and made such surfaces more reliable and consistent for osseointegration. This effect, however, was not detectable in either of the BG coating groups. In the rather thick BG coatings investigated here, a foreign body reaction was detectable to different extents, which could be speculated as the reason for the mechanically reduced osseointegration. Thus, bioactive surface treatments can indeed help to increase endogenous osseointegration capacity in healthy metaphyseal bone (such as in this sheep animal model) but does not in all cases lead to a highly consistent osseointegration. The best results were observed with the rather thin (nanometer sized) coating while both relatively thick BG coatings did not prove to be beneficial under the conditions of the present experiment. It would be interesting to analyze in future if these effects are consistent in more challenging scenarios and to consider different BG compositions, coating microstructures and thicknesses.
